# Antimicrobial and Genotoxicity Effects of Zero-valent Iron Nanoparticles

**DOI:** 10.5812/jjm.10054

**Published:** 2014-05-01

**Authors:** Elham Barzan, Sedigheh Mehrabian, Saeed Irian

**Affiliations:** 1 Department of Microbiology, Faculty of Science, Urmia Azad University, Urmia, IR Iran; 2 Department of Cell and Molecular Biology, Faculty of Biological Sciences, Kharazmi University, Tehran, IR Iran

**Keywords:** Nanoparticles, Bronchial Provocation Tests, Mutagenicity

## Abstract

**Background:**

In a world of nanotechnology, the first concern is the potential environmental impact of nanoparticles. An efficient way to estimate nanotoxicity is to monitor the responses of bacteria exposed to these particles.

**Objectives:**

The current study explored the antimicrobial properties of nZVI (zero-valent Iron nanoparticles) on the Gram-negative bacterial systems *Erwinia amylovora*, *Xanthomonas oryzae* and the Gram-positive bacterial systems *Bacillus cereus* and Streptomyces spp. The genotoxicity potential of nZVI was also assayed.

**Materials and Methods:**

The toxicity of nZVI was tested by two different methods: Growing bacteria in liquid (broth dilution) and agar media (challenge test) containing different nZVI concentrations for 24-72 hours. The genotoxicity of nZVI was assessed using the preincubation version of the Ames test.

**Results:**

The lowest concentrations of nZVI that inhibited the visible growth (MIC) of *E. amylovora*, *X. oryzae*, *B. cereus *and *Streptomyces* spp. were 625, 550, 1250 and 1280 ppm, respectively. The minimum bactericidal concentration (MBC) for *E. amylovora* and *X. oryzae* were 10,000 and 5,000 ppm of nZVI, respectively. MBC was not observed for the Gram positive bacteria. No bacteriostatic and bactericidal effects were observed for oxidized nZVI. Mutant frequency did not increase according to the vehicle control at the concentrations assayed, indicating a lack of mutagenicity associated with nZVI.

**Conclusions:**

nZVI nanoparticles are not mutagenic at low concentrations, therefore they can be used without detrimental effects on soil bacteria.

## 1. Background

Nanotechnology is a new and rapidly growing scientific field. Recent advances in this field have resulted in the production of different kinds of metal and metal oxide nanoparticles with antibacterial effects (1). These are particles with an average dimension range of 1-100 nm (2), and are divided into three groups of natural, incidental, and engineered nanoparticles (3). An important property of these particles is their greater surface area which is reflected by their increased reactivity (4). Nanomaterials have had several industrial applications in the production of commercial products in the areas of cosmetics, health, clothing, electrical, agriculture and even food and medicine (5).

The search for a simple, inexpensive and efficient method of eliminating contaminations has drawn a great deal of attention to iron nanoparticles. In 1996, zero-valent iron nanoparticles (nZVI) were introduced as an environmental remediation agent. Later, two different groups used nZVI in the permeable reactive barrier (PRB) technology (6, 7). PRB technology, which makes use of zero-valent iron, has been used for underground water remediation for more than 20 years (8). nZVI with an average dimension range of 50-300 nm and highly reactive is presently being used for environmental remediation (9). 

Iron nanoparticles, which are highly reactive and quickly oxidized by oxygen, are also being used for environmental remediation of heavy metals such as cadmium, mercury, silver and nickel, as well as chlorine, in particular in soil and water remediation (7). It has been shown that nZVI is capable of eliminating pesticides such as DDT and Lindane in addition to inorganic ions such as dichromate, arsenic, perchlorate and nitrate (10). Synthesis of nZVI is a relatively simple procedure and can be obtained by Fe^3+^ reacting with sodium borohydride in an aqueous solution (11).

4Fe^3+^ + 3BH^4-^ + 9H_2_O→ 4Fe^0^ + 3H_2_BO^3-^ + 12 H^+^ + 6H_2_

Man is surrounded by a variety of carcinogens present in the environment. These agents by inducing changes in the sequence of the genetic material (mutation) may cause cancer. Hence, the identification of such mutagens is an important first step in improving human health, in particular, if they result in germ line mutations which can be inherited through generations. To this end, improving inexpensive, quick and simple methodologies to identify potential mutagens are of great health value (12). One such methodology makes use of bacteria, mainly due to their great rate of proliferation and other chemical and genetic properties. To date, there are several bacterial strains known to show sensitivity to a wide range of mutagens (13). In addition, the inability to synthesize cytochrome P450 mediated products makes them suitable for the analysis of potential mutagens (14).

A well established method of determining the potential mutagenic nature of a substance in a bacterial system is the Ames test which uses different strains of *Salmonella typhimurium*. Each of these strains carries a specific mutation in its histidine operon, and thus is incapable of synthesizing the amino acid histidine (His), whereas the wild type strain is prototroph (His^+^), and can do so when provided with ammonium phosphate and a suitable carbon source such as glucose. Strains of *S. typhimurium* used in the Ames test are auxotroph (His^-^), and when grown in solid media containing a minimum amount of histidine along with a potential mutagen, only the bacteria with a reverse mutation in the His gene (revertants) are capable of growth and colony formation. Therefore, the Ames test operates by assaying for the presence of revertants, and its accuracy in detecting potential mutagens has been demonstrated (14, 15). In addition to His auxotrphy, the TA100 strain carries both *rfa* and *uvrB-bio* mutations. The *rfa* mutation changes the properties of the bacterial cell wall by resulting in a defective lipopolysaccharide (LPS) layer that coats the bacterial surface, making the bacteria more permeable to larger molecules, while the *uvrB-bio* mutation makes the strain sensitive to UV light (unable to repair damaged DNA) and confers a biotin auxotrophy. Finally, this strain carries a mutation that enhances R-factor plasmid pKM101 activity which confers ampicillin resistance (16).

## 2. Objectives

 The present study investigated the antimicrobial effect of nZVI on four different agricultural soil bacteria, and its anti-mutagenic effect using the Ames test.

## 3. Materials and Methods

### 3.1. Nanoparticles, Chemicals and Bacterial Strains

nZVI with dimensions of 20-50 nm were provided in a black color non-homogenate solution by Pasargad Lotus Nanochemical Company (Iran). All other chemicals were purchased from Merck (Germany). Bacterial strains *Erwinia amylovora*, *Xantomonas oryzae*, *Bacillus cereus* and *Streptomyces* spp. were provide by the University of Jihad Agricultural Research (Tehran-Iran), and stored at 4˚C. *S. typhimurium* TA100 was kindly provided by Professor Ames and stored at -80˚C.

### 3.2. Determining the Minimum Inhibitory Concentration

To determine the minimum concentration of nZVI causing bacterial growth inhibition, tubes containing 1mL of Mueller Hinton Broth for the three bacterial strains *E. amylovora*, *X. oryzae* and *B. cereus*, and a tube containing 1mL of Sabouraud dextrose broth for *Streptomyces* spp. were initially prepared. Then, 1 mL of different concentrations of nZVI (1000-10000 ppm) was added to the tube containing 1 mL of a bacterial culture, followed by a 12 serial dilution to the total of 13 samples. These cultures for the three bacterial strains *E. amylovora*, *X. oryzae* and *B. cereus* were incubated at 28-30˚C, while those of Streptomyces spp. were incubated at 37˚C in a shaking incubator. MIC was then determined for each tube in 24 hours intervals.

### 3.3. Determining the Minimum Bacterial Concentration

To determine the minimum concentration of nZVI causing bacterial death, following the preparation of Mueller Hinton Agar plates for bacterial strains *E. amylovora*, *X. oryzae* *B. cereus*, and Sabouraud dextrose agar plates for *Streptomyces* spp., several microliters of the samples before and after the MIC sample was inoculated onto these plates. These plates were incubated at the optimum growth conditions, specified for each bacterium. MBC was then determined for plates lacking bacterial growth.

### 3.4. Challenge Test

The test aimed to determine bacterial growth, or lack of growth in the presence of different concentrations of nZVI. For this purpose, 100 μL of each of the nZVI dilutions was added to 100 μL of a bacterial culture, separately. These mixtures were then added to 3 mL of NaCl-containing top agar, and spread on the appropriate agar plates. These plates were incubated at the optimum growth conditions, specified for each bacterium.

### 3.5. Confirmation of S. typhimurium TA100 Genotype

Prior to performing the anti-mutagenic assay, *S. typhimurium* TA100 mutation was confirmed. The strain was grown on a nutrient agar plate, and the genotype was checked routinely for its histidine auxotrophy, deep rough (*rfa*) character, UV sensitivity (*uvrB* mutation,) and the presence of the R factor plasmid. Samples were stored at -80˚C. This strain requires histidine to grow, and in the absence of an external histidine source, cells cannot grow to form colonies. The presence of the *uvrB* mutation makes the strain sensitive to UV light. The *rfa* mutation changes the properties of the bacterial cell wall and causes a defective lipopolysaccharide (LPS) layer that coats the bacterial surface, making the bacteria more permeable to larger molecules. The *rfa* mutation is indicated by sensitivity to crystal violet. The presence of R factor plasmid in this strain shows that they are resistant to ampicillin (14-16).

### 3.6. Confirmation of the Appropriate Concentration of nZVI for the Ames Test

*S. typhimurium* TA100 was cultured in the presence of different concentrations of nZVI. This assay also included positive (bacteria without nZVI) and negative (nZVI without bacteria) controls.

### 3.7. Mutagenicity Assay Using S. typhimurium TA100

In the first stage of the assay, 0.1 mL of the test material (i.e. nZVI) was added to 0.1 mL of a fresh overnight (16 hours) *S. typhimurium* TA100 culture at a concentration of 1 × 10^9^ CFU/ml, followed by the addition of 0.1 mL of histidine-biotin solution in a sterilized top agar-containing tube. This tube was then mixed on a shaker for three seconds, and evenly spread on an agar plate containing minimal medium, in duplicates. The plates were incubated at 37˚C for 48 hours. Positive and negative controls were also included in the assay. The negative control contained bacteria, a histidine-biotin solution and ddH_2_O, and it was used for the purpose of monitoring the rate of spontaneous mutations. The positive control included bacteria, a histidine-biotin solution and the well known mutagen, sodium azide. These were also plated on agar plates containing glucose and minimal medium, and the plates were incubated at 37˚C for 48 hours. Following the incubation, the number of revertant colonies was counted in all plates.

Mutagenicity was determined on the basis of Q_m_ value that is the ratio of the number of revertant colonies in the test plates to those of the negative control. A Q_m_ value of ≥ 2 is indicative of a mutagen, while a value of 1.7 to 1.9 indicates a potential mutagen and a value of ≤ 1.6 is considered non-mutagenic (17, 18).

## 4. Results

### 4.1. MIC and MBC Tests

MIC and MBC results for both nZVI and oxidized nZVI have been provided in Table 1. These results indicated that nZVI, at certain concentrations, inhibited bacterial growth, while oxidized nZVI did not do so at any of the nZVI concentrations.

**Table 1.** Comparison of MIC and MBC Values for Zero-valent Iron Nanoparticle (nZVI) and Oxidized nZVI ^a^, ^b^

Bacteria	nZVI, ppm		Oxidized nZVI, ppm/mL	
	MIC	MBC	MIC	MBC
***Erwinia amylovorac***	625	10000	-	-
***Xantomonas oryzaec***	550	5000	-	-
***Bacillus cereusc***	1250	-	-	-
***Streptomyces*** **spp.**	1280	-	-	-

^a^ Abbreviations: MBC, minimum bactericidal concentration ; MIC, minimum inhibitory concentration.

^b^ Average of three MIC values; average of three MBC values; MIC value in Mueller Hinton broth, and MBC value in Mueller Hinton agar media; MIC value in Sabouraud dextrose broth, and MBC value in Sabouraud dextrose agar media; no inhibitory zone was detected at examined concentrations.

### 4.2. Challenge Test

**Figure 1.**
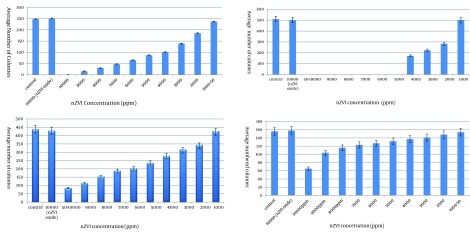
Comparison of Bacterial Growth in the Presence of Different Concentrations of Zero-valent Iron Nanoparticle (nZVI) and Oxidized nZVI With Those of the Control *A. Erwinia amylovora*; *B. Xantomonas oryzae*. * C.Bacillus cereus*. *D. Streptomyces *spp.

Results on the growth of the contents of MIC tubes on agar plates (challenge test) indicated that nZVI at concentrations of 1000 ppm and 5000 ppm inhibited the growth of *E. amylovora* and *X. oryzae*, respectively. While no such growth inhibition was detected for *B. cereus* and *Streptomyces* spp. at any of the nZVI concentrations. Finally, no bacteriostatic or bactericidal effect was detected for oxidized nZVI at any of the nZVI concentrations (Figure 1).

### 4.3. Mutagenicity Assay

Results of *S. typhimurium* TA100 genotype confirmation are provided in Table 2, and the appropriate concentration of nZVI for the Ames test was determined at 1000ppm. Results of nZVI mutagenicity have been provided in Table 3. As indicated in Table 3, nZVI does not possess a significant (P = 99%) mutagenicity effect when compared to the negative control.

**Table 2.** Results of *S. typhimurium* TA100 Genotype Confirmation

Bacteria	Genotype		
	R-factor ^a^	*uvrB* Mutation ^b^	*rfa* Mutation ^c^
***S. typhimurium*** ** TA100**	+	+	+

^a^ Resistance to ampicillin, indicative of the presence of the R-factor.

^b^ Sensitivity to ultraviolet radiation, indicative of *uvrB* mutation.

^c^ Sensitivity to crystal violet, indicating *rfa* mutation.

**Table 3.** Results of the Mutagenic Effect of Zero-valent Iron Nanoparticle ^a^

Samples	Number of *S. typhimurium* TA100 Revertant Colonies
**Negative control (ddH**_**2**_ **O)**	10 ± 0.577
**Positive control (sodium azide)**	411 ± 34.18
**nZVI**	11 ± 1.2

^a^ Data are presented in Mean ± SE.

## 5. Discussion

Results of the challenge test for the bacteria *E. amylovora* and *X. oryzae* in the presence of nZVI showed the growth inhibition. Bacteriocidic effect was also detected at higher nZVI concentrations (> 5000 ppm) for the two strains. In the current investigation, concentration of the nanoparticle appeared to have an important role in bacterial inactivation, a finding that is in line with the other reports (19-21).* B.* *cereus* and *Streptomyces* showed a greater resistance to nZVI compared to Gram-negative bacteria, and growth inhibition was observed, while no bactericidal effect was detected. This may be explained by the presence of the thick (20-80 nm) peptidoglycan layer in the cell walls of Gram positive bacteria, making them more resistant to the nanoparticle (22).

The underlying mechanism of nZVI cytotoxicity is still a matter of debate (23). Researches have shown that a direct nZVI-cell contact has an important role in nZVI cytotoxicity (24, 25). The most important mechanism underlying the bactericidal effect of antibiotics and other drugs is the induction of oxidative stress through generation of reactive oxygen species (ROS) (26) such as superoxide (O_2_•^-^), hydroxyl radicals (OH•) and hydrogen peroxide that eventually lead to cellular protein and DNA damage (27). Park et al. (2009) demonstrated a ROS-mediated bactericidal effect of silver nanoparticles (28). Lee et al. (2008) concluded that *E. coli* inactivation in the presence of nZVI might be mediated by the penetration of particles with an average dimension of 10-80 nm through the cell wall, followed by their interaction with the intracellular oxygen, resulting in oxidative stress and eventually bacterial cell death (1). Therefore, it is likely that such a mechanism may also be operating during the cytotoxic effect of nZVI with an average dimension of 20-50 nm used in the present investigation. The oxidized nZVI used in the current study did show a sign of bactericidal effect towards the tested bacterial strains. A reduction in the bactericidal effect of nZVI, following its oxidation, has been attributed to the formation of an oxidized iron layer on the nanoparticle (1). It is noteworthy that the black color of nZVI turns yellow, following oxidation (29). The important role played by the concentration of nanoparticles in their bacteriostatic and bactericidal effect, observed in this study, has also been reported by the others working on the antibacterial effects of silver and ZnO nanoparticles (19, 21). The Ames test has long been used as an accurate method to identify potential mutagens, in such a way that over 70% of the mutagens identified thus far have used *Salmonella* spp. (30). In the present study, using *S. typhimurium* TA100, nZVI at a concentration of 1000 ppm showed to have no mutagenic effect. This concentration of nZVI, however, did not have any antibacterial effects towards *S. typhimurium* TA100. The Ames test has also shown that the iron oxide nanoparticles have no mutagenic effect (31). Similar results have been observed by the others analyzing the in vivo genotoxicity effect of silver nanoparticles (32). nZVI nanoparticles are not mutagenic at low concentrations, and hence can be used without any detrimental effects on soil bacteria.
